# Benefits of Levothyroxine Replacement Therapy on Nonalcoholic Fatty Liver Disease in Subclinical Hypothyroidism Patients

**DOI:** 10.1155/2017/5753039

**Published:** 2017-04-04

**Authors:** Lu Liu, Yong Yu, Meng Zhao, Dongmei Zheng, Xu Zhang, Qingbo Guan, Chao Xu, Ling Gao, Jiajun Zhao, Haiqing Zhang

**Affiliations:** ^1^Department of Endocrinology, Shandong Provincial Hospital Affiliated to Shandong University, Jinan, Shandong 250021, China; ^2^Shandong Clinical Medical Center of Endocrinology and Metabolism, Jinan, Shandong 250021, China; ^3^Institute of Endocrinology and Metabolism, Shandong Academy of Clinical Medicine, Jinan, Shandong 250021, China; ^4^Department of Sonography, Shandong Provincial Hospital Affiliated to Shandong University, Jinan, Shandong 250021, China; ^5^Scientific Center, Shandong Provincial Hospital Affiliated to Shandong University, Jinan, Shandong 250021, China

## Abstract

*Objectives*. To evaluate the effect of levothyroxine (LT_4_) replacement therapy on nonalcoholic fatty liver disease (NAFLD) in subclinical hypothyroidism (SCH) patients. *Methods*. This study was a post hoc analysis of a randomized controlled trial and involved 33 significant and 330 mild SCH patients. All of the significant SCH patients received LT_4_ supplement. The mild SCH patients were grouped as LT_4_ treated or not. After 15 months of follow-up, prevalence of NAFLD in each group was reevaluated. Subgroup analysis was conducted in mild SCH patients with dyslipidemia. *Results*. After treatment with LT_4_, the prevalence of NAFLD in significant SCH patients reduced from 48.5% to 24.2% (*p* = 0.041). In mild SCH patients, prevalence of NAFLD and serum alanine aminotransferase (ALT) was not significantly affected by LT_4_ supplementation. Nonetheless, mild SCH patients with dyslipidemia who received LT_4_ treatment experienced decreases in the prevalence of NAFLD and serum ALT levels (*p* < 0.05 for both). In contrast, these parameters remained comparably stable in patients who were not treated. *Conclusion*. LT_4_ supplementation has benefits on NAFLD in significant SCH patients or mild SCH patients with dyslipidemia. For NAFLD patients with SCH, appropriate supplementation of LT_4_ may be an effective means of controlling NAFLD. The original trial was registered with ClinicalTrials.gov (NCT01848171).

## 1. Introduction

Nonalcoholic fatty liver disease (NAFLD) is defined as extrahepatic accumulation of fat in the absence of excess alcohol consumption. This condition is the most common cause of asymptomatic abnormal liver function tests [1], with its prevalence worldwide estimated to be 20% to 30% [2, 3], and the burden of this disease is translating to an increasing demand for liver transplantation [4]. Moreover, NAFLD is closely associated with components of metabolic syndrome, including visceral obesity, dyslipidemia, and insulin resistance and is indicative of an increased risk of cardiovascular disease and type 2 diabetes [5, 6]. Despite gratifying progress in the etiology of NAFLD in recent years, the mainstay of therapy remains weight loss through modification of diet and lifestyle [7]. However, even with an intensified lifestyle modification program, not all patients could achieve remission of NAFLD [8], underscoring the need for more specific targeted therapies.

Subclinical hypothyroidism (SCH) is an early manifestation of thyroid underactivity, which is characterized by elevated serum thyroid-stimulating hormone (TSH) levels, whereas serum-free thyroxine (FT_4_) levels are within the normal range [9]. It is a common medical condition with a prevalence ranging from 4% to 20% of adults, and the prevalence is progressively increasing [10, 11]. As stated by *Lancet*, “The world faces a burden of thyroid disease that has reached epidemic proportions” [12]. Remarkably, a growing body of evidence has led to the hypothesis that SCH is an independent risk factor of NAFLD [13–16]. A cross-sectional study reported that the prevalence of NAFLD in SCH patients was significantly increased compared with that of subjects with euthyroidism (29.9% versus 19.5%, *p* < 0.001) [13]. Moreover, population-based studies have demonstrated that increased TSH levels indicate an elevated incidence of NAFLD [14]. In addition, our group observed that TSH could directly regulate hepatic cholesterol and triglyceride metabolism and subsequently exacerbate the accumulation of fat in the liver [17–19]. Levothyroxine (LT_4_) is the standard medication for treatment of hypothyroidism and is associated with a significant decrease in body weight and serum lipids [20, 21]. Therefore, does the treatment of SCH patients to normalize elevated serum TSH levels via appropriate LT_4_ supplementation yield any benefits on NAFLD? No interventional study is currently focusing on this issue.

We performed this post hoc analysis of a randomized controlled trial to evaluate the effect of SCH treatment on NAFLD, aiming to provide a new choice for the treatment of NAFLD. In addition, SCH is closely associated with disturbances in lipid metabolism [22, 23], and dyslipidemia is a risk factor of NAFLD [24–26]. We further conducted subgroup analysis to assess the effect of LT_4_ supplementation on NAFLD in mild SCH patients with dyslipidemia.

## 2. Materials and Methods

### 2.1. Study Design and Patients

The design of the original study has been described elsewhere [21]. Briefly, the original study was an open-label, randomized, controlled trial designed to assess the effects of LT_4_ replacement therapy on lipid profiles in SCH patients. Subjects were recruited from Ningyang County, Shandong Province, China. All registered Chinese civilians aged 40 years or older who had lived in Ningyang County for at least five years were invited by telephone or door-to-door visits to undergo the screening program for SCH, which began in July 2013. Subjects diagnosed with SCH (TSH ≥ 4.2 mIU/L with normal serum FT_4_ confirmed on the basis of at least two hormonal assays with a three-month interval [27]) and in the absence of previous or ongoing treatment for hypothyroidism were included in the trial. Exclusion criteria were as follows: (1) pregnancy or breast-feeding, (2) complications or conditions that affect thyroid status or lipid metabolism, (3) taking any medicine that affects the thyroid or lipid metabolism in the previous three months, and (4) obviously poor compliance. Finally, 415 SCH patients, including 37 significant SCH (TSH ≥ 10 mIU/L) and 378 mild SCH (TSH of 4.2–10 mIU/L), were enrolled in the trial. All of the participants completed abdominal ultrasonography at enrollment.

All patients provided informed written consent. The study protocol conformed to the ethical guidelines of the 1975 Declaration of Helsinki, was approved by the Ethics Committee of the Provincial Hospital affiliated to Shandong University, and was registered with ClinicalTrials.gov (NCT01848171).

### 2.2. Randomization

According to the guideline for hypothyroidism cosponsored by the American Association of Clinical Endocrinologists and the American Thyroid Association [27], significant SCH patients were all treated with LT_4_ (Euthyrox, 50 *μ*g per tablet, Merck Serono, Darmstadt, Germany). Mild SCH patients were randomized in a 1.5 : 1 fashion to LT_4_ replacement therapy or no treatment. Randomization was performed through the use of computer-generated random numbers. Neither patients nor investigators involved in the study were masked to treatment allocation.

### 2.3. Intervention

For patients treated with LT_4_, the initial dosage was 25 *μ*g/day. Treatments were administered with water between 30 and 60 minutes prior to eating breakfast [27]. Thyroid function was re-evaluated six weeks later based on a test of serum TSH and FT_4_ levels, and the dosage of LT_4_ was adjusted accordingly. The adjustment continued until euthyroidism (TSH of 0.27–4.2 mIU/L with a normal serum FT_4_ level) was reached, and the dosage that achieved euthyroidism was subsequently maintained. Finally, the median LT_4_ dosage was 75 *μ*g daily for significant SCH patients and 50 *μ*g daily for mild SCH patients who received LT_4_ replacement therapy. All recruited subjects were interviewed every three months. Updates on patient status were recorded at each visit, including any diseases, drugs, operations, and obvious changes in lifestyle (e.g., vegetarian diet or intense exercise). In addition, patients who received LT_4_ replacement therapy were advised to return any unused tablets at each visit to allow monitoring of the compliance with the allocated medication. All of the participants received follow-up for 15 months.

### 2.4. Study Endpoints

The primary endpoint of the original trial was serum total cholesterol (TC) concentration change from baseline to end-of-study. The secondary endpoints were the changes in other serum lipid parameters during the study course, including low-density lipoprotein cholesterol (LDL-C), high-density lipoprotein cholesterol (HDL-C), and triglyceride (TG). In the present post hoc analysis, the primary endpoint was the effect of LT_4_ replacement therapy on the prevalence of NAFLD. Secondary endpoints were changes in serum alanine aminotransferase (ALT) and aspartate aminotransferase (AST) levels.

### 2.5. Follow-Up Assessments

All subjects completed a self-report questionnaire, including information on demographic characteristics, medical history, and lifestyle, by trained interviewers at recruitment. Clinical assessment was performed at baseline and end-of-study and included measurements of weight, height, waist circumference (WC), blood pressure, and liver ultrasound. Weight (kilograms) and standing height (meters) were measured between 8:00 a.m. and 10:00 a.m. after a minimum 10-hour fasting in light clothing and without shoes. Body mass index (BMI) was calculated by dividing weight by the square of the height. The methods for determining WC and blood pressure were similar to those used in the national survey of the prevalence of diabetes in 2010 [28].

Liver ultrasound was performed using a 3.75 MHz curvilinear probe (model CC-15M71-MA; Toshiba, Tokyo, Japan) by the same experienced radiologist throughout the study. The radiologist was blind to the study design and laboratory values of the participants. The diagnostic criteria for fatty liver by ultrasonography included the following items: (1) diffuse enhancement of near field echogenicity in the hepatic region (compared with that in the kidney and spleen region) and gradual attenuation of the far field echogenicity, (2) unclear display of intrahepatic lacuna structure, (3) mild to moderate hepatomegaly with a round and blunt border, (4) color doppler ultrasonography shows a reduction of the blood flow signal in the liver or it is even hard to display, but the distribution of blood flow is normal, and (5) blurred or nonintact display of the envelope of the right liver lobe and diaphragm. Fatty liver was diagnosed if item 1 and any one or more of items 2–4 are matched [29, 30]. NAFLD was diagnosed based on the criteria suggested by the Chinese Society of Hepatology, Chinese Medical Association [30], as fatty liver confirmed by ultrasonic imaging and in the absence of the following: (1) excess alcohol consumption (>70 g per week for women and >140 g per week for men), (2) diagnosis of viral hepatitis, (3) usage of hepatotoxic drugs, and (4) other diseases that might cause fatty liver [31].

Venous blood samples were drawn between 8:00 a.m. and 10:00 a.m. after a minimum 10-hour fasting. Serum-free triiodothyronine (FT_3_), FT_4_, and TSH levels were measured by chemiluminescence methods (Cobas E601; Roche, Basel, Switzerland). The serum lipid profiles, fasting plasma glucose (FPG), liver enzymes, and renal functions were quantified using a Beckman Chemistry Analyzer AU5800 System (Beckman Coulter, Tokyo, Japan). Non-HDL cholesterol (non-HDL-C) was calculated by subtracting HDL-C from TC. The examinations of these variables were all completed at the clinical laboratory of the Shandong Provincial Hospital. The laboratory reference ranges were 3.1–6.8 pmol/L (2.01–4.42 pg/mL) for FT_3_, 12–22 pmol/L (0.94–1.72 ng/dL) for FT_4_, 0.27–4.2 mIU/L for TSH, 9–50 IU/L for ALT, and 15–40 IU/L for AST. Diabetes was diagnosed based on the World Health Organization (WHO) 1999 criteria, as FPG concentration ≥ 7.0 mmol/L and/or self-reported history of type 2 diabetes [32].

### 2.6. Statistical Analysis

Quantitative data are expressed as the mean ± standard deviation or median (interquartile range) according to their distributions, and categorical data are presented as a number (percentage). Comparisons of variables among the groups were performed using one-way ANOVA, independent Student's *t*-test, or the Mann-Whitney *U* test. A paired-samples *t*-test or Wilcoxon paired rank test was used for within-group comparisons (baseline and end-of-study variables in each group). Differences in categorical data were evaluated by chi-squared test. A two-tailed value of *p* < 0.05 was regarded as significant. All statistical analyses were performed using SPSS version 22.0 for Windows (Chicago, IL, USA). All authors had access to the study data and reviewed and approved the final manuscript.

Given the high prevalence of dyslipidemia in SCH patients and the role of dyslipidemia as an important risk factor of NAFLD, we performed subgroup analysis to evaluate the effect of LT_4_ replacement therapy on NAFLD in mild SCH patients with dyslipidemia at baseline. Dyslipidemia was defined as abnormalities in the serum levels of lipids, including TG ≥ 1.70 mmol/L or TC ≥ 6.22 mmol/L or LDL-C ≥ 4.14 mmol/L or HDL-C < 1.04 mmol/L for men and <1.30 mmol/L for women [33].

## 3. Results

### 3.1. Baseline Characteristics

**Figure 1:**
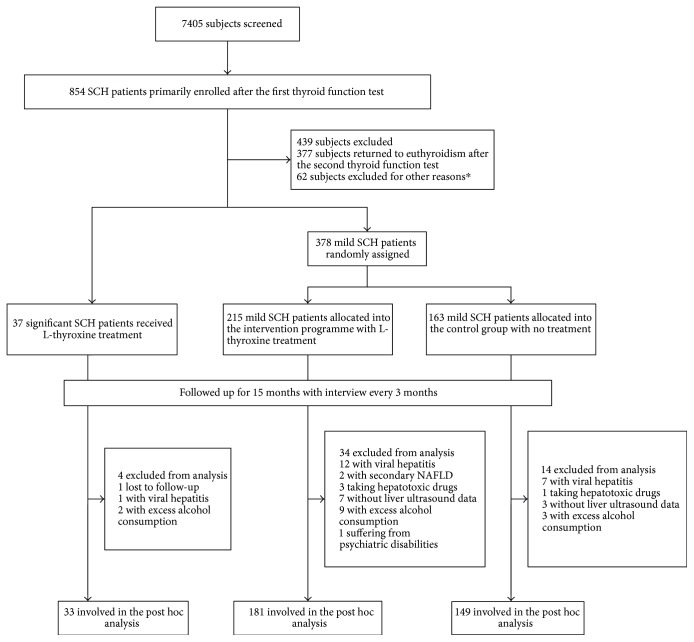
Flow diagram. ^∗^Reasons for exclusion are given in Materials and Methods. SCH: subclinical hypothyroidism; NAFLD: nonalcoholic fatty liver disease.

Based on the self-reported medical history, subjects with viral hepatitis, secondary causes of NAFLD, and use of hepatotoxic drugs, as well as with excess alcohol consumption or incomplete abdominal ultrasound data, were further excluded from the analysis (*n* = 52). Finally, this post hoc analysis involved 363 participants, including 33 significant SCH patients and 330 mild SCH patients (Figure 1). Among the mild SCH patients, 181 were treated with LT_4_ (mild SCH-LT_4_ group) and 149 were not treated (mild SCH-control group).  Table 1 presents the baseline characteristics of the participants in three groups. The cohort was middle aged with a predominance of females, and the majority of the participants were overweight or obese (BMI ≥ 24 kg/m^2^), [34]. Notably, approximately two-thirds of the subjects also had dyslipidemia. Nine (27.3%) out of the 33 significant SCH patients suffered from diabetes at enrollment. The prevalence of diabetes in the mild SCH-LT_4_ group and the mild SCH-control group were 13.8% and 16.8% (*p* = 0.455), respectively. The prevalence of NAFLD in the significant SCH patients was 48.5%. In mild SCH patients, the prevalence of NAFLD in the LT_4_ group and the control group was 44.2% and 39.6%, respectively. The prevalence of NAFLD in the studied SCH patients was considerably increased compared with that reported in general population which was estimated at 20% to 30% [2, 3]. The baseline demographic, anthropometric, biochemical, and clinical variables were evenly matched in the two arms of mild SCH patients.

### 3.2. Efficacy of LT_4_ on Thyroid Function

Thyroid functions throughout the study were shown in Supplementary Table 1 available online at https://doi.org/10.1155/2017/5753039. At end-of-study, all of the SCH patients that received LT_4_ treatment, including the significant SCH patients and the mild SCH patients in the mild SCH-LT_4_ group, achieved normalization of thyroid function, with a significant decrease in TSH level and a significant increase in FT_4_ level compared with that at baseline (*p* < 0.05 for all). In the mild SCH-control group, serum FT_4_ level remained stable throughout the course of the study. Although the serum TSH level in the mild SCH-control group exhibited a naturally slight decrease at end-of-study, it was still higher than the normal range (0.27–4.2 mIU/L). Serum FT_3_ was not significantly altered throughout the study in each group.

### 3.3. Primary Endpoint: Prevalence of NAFLD

Within the significant SCH group, the prevalence of NAFLD was reduced from 48.5% to 24.2% (*p* = 0.041) during the course of the study (Table 2). The prevalence of NAFLD in both arms of mild SCH patient showed moderate but not statistically significant reduction. In the mild SCH-LT_4_ group, the prevalence of NAFLD decreased from 44.2% to 35.9% (*p* = 0.108). The prevalence of NAFLD in the mild SCH-control group also reduced (39.6% to 34.9%, *p* = 0.402). As can be seen, the reduction in the prevalence of NAFLD tended to be smaller in the mild SCH-control group compared with that in the mild SCH-LT_4_ group (4.7% versus 8.3%), though the difference did not reach the statistically significant level (*p* = 0.432) (Table 2). These results indicated that clinicians should be alert to the possibility of SCH in NAFLD patients. Regarding NAFLD patients with significant SCH, LT_4_ supplementation may be an effective means for controlling NAFLD.

### 3.4. Secondary Endpoint: Serum Liver Enzymes

Baseline and end-of-study data for liver function in each group are shown in Table 2. After LT_4_ treatment, significant SCH patients experienced a decrease of 5.61 IU/L in serum AST (*p* < 0.001) and a moderate but not statistically significant decrease in serum ALT.

Patients in the mild SCH-LT_4_ group showed a marginally significant reduction in serum ALT from 19.09 IU/L to 17.95 IU/L (*p* = 0.087), whereas serum ALT in the mild SCH-control group was quite stable in the study. Additionally, although there were reductions in serum AST within the two groups of mild SCH patients, the mild SCH-LT_4_ group reported a remarkably greater reduction than the mild SCH-control group (−4.86 versus −3.29 IU/L, *p* = 0.046).

### 3.5. Changes in Metabolic Characteristics in the Cohort

Details are presented in the Supplementary Material (Supplementary results, Supplementary Table 2, and Supplementary Table 3).

### 3.6. Subgroup Analysis: Mild SCH Patients Combined with Dyslipidemia

The prevalence of dyslipidemia in the studied mild SCH patients was as high as 62.7% (207 of 330). In addition, dyslipidemia is a risk factor of NAFLD, so we performed subgroup analysis to evaluate the effect of LT_4_ supplementation on NAFLD in mild SCH patients with dyslipidemia. The 207 mild patients with dyslipidemia at baseline were involved in this subgroup analysis and were grouped as the sub-LT_4_ group and subcontrol group according to whether they received LT_4_ replacement therapy. Baseline characteristics for these patients are displayed in Supplementary Table 4. The demographic, anthropometric, biochemical, and clinical characteristics were statistically matched between the sub-LT_4_ group and subcontrol group at baseline.

#### 3.6.1. Morbidity of NAFLD

**Figure 2:**
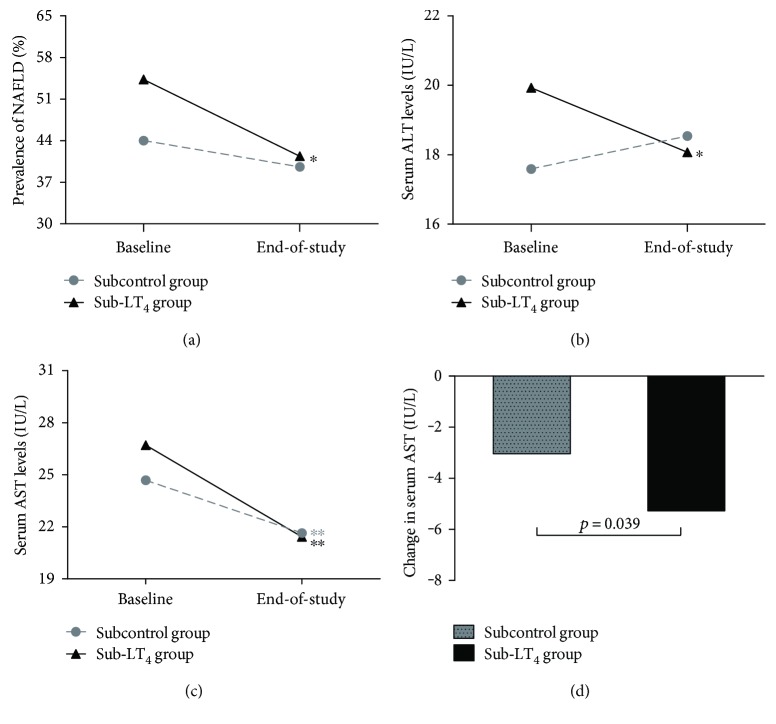
Effects of LT_4_ replacement therapy on prevalence of NAFLD and serum liver enzymes in mild SCH patients with dyslipidemia. (a) Prevalence of NAFLD for each group at baseline and end-of-study. (b–d) Changes in serum liver enzymes for each group. Data are presented as means. (b) Serum ALT at baseline and end-of-study. (c) Serum AST at baseline and end-of-study. (d) Reductions in AST during the course of study for each group. ^∗^*p* < 0.05 and ^∗∗^*p* < 0.01 for comparison between baseline and end-of-study, respectively. SCH: subclinical hypothyroidism; NAFLD: nonalcoholic fatty liver disease; LT_4_: levothyroxine; ALT: alanine aminotransferase; AST: aspartate aminotransferase.

The prevalence of NAFLD was statistically comparable between the sub-LT_4_ group and subcontrol group at baseline (*p* = 0.139). Within the sub-LT_4_ group, the prevalence of NAFLD decreased from baseline to end-of-study (54.3% to 40.5%, *p* = 0.035) (Figure 2(a)). Although the prevalence of NAFLD also decreased in the subcontrol group during the study, the reduction did not reach significance (44.0% to 39.6%, *p* = 0.548) (Figure 2(a)). In addition, we further analyzed the remission rate of NAFLD among patients suffering from NAFLD at baseline and the incidence rate of NAFLD among patients without NAFLD at baseline. In the sub-LT_4_ group, 26 (41.3%) of 63 patients with NAFLD at baseline reverted to the normal liver at end-of-study, and the level tended to be higher compared with that of the subcontrol group (35.0%, *p* = 0.525). Among patients without NAFLD at baseline, the incidence of NAFLD at end-of-study in the sub-LT_4_ group was 18.9%, which is slightly lower compared with that in the subcontrol group (19.6%, *p* = 0.924) (Supplementary Table 5).

#### 3.6.2. Serum Liver Enzymes

Regarding liver enzymes, there was no difference in serum ALT or AST levels between the sub-LT_4_ group and the subcontrol group at baseline (*p* > 0.05 for all). Within-group comparisons revealed that serum ALT decreased from 19.93 IU/L to 18.07 IU/L (*p* = 0.043) in the sub-LT_4_ group but increased marginally in the subcontrol group (17.59 IU/L to 18.54 IU/L, *p* = 0.282) (Figure 2(b)). Moreover, serum AST declined in both groups during the study, but the reduction in the sub-LT_4_ group was significantly greater than that in the subcontrol group (−5.28 IU/L versus −3.04 IU/L, *p* = 0.039) (Figures 2(c) and 2(d)).

#### 3.6.3. Metabolic Response

Statistically significant reductions (*p* < 0.05) in mean body weight and BMI were noted in patients treated with LT_4_, whereas patients who were not treated exhibited comparatively stable body weight and BMI (Table 3). Moreover, there was a trend toward a greater decrease in body weight in patients who experienced remission of NAFLD (*n* = 40) compared with those without remission of NAFLD (*n* = 63) (−1.65 versus −0.60 kg, *p* = 0.212), and the same trend of decrease was reported for BMI (−0.36 versus −0.17 kg/m^2^, *p* = 0.586). These results indicated that improvement of NAFLD in patients treated with LT_4_ might be associated with weight loss. Regarding serum lipids, patients treated with LT_4_ experienced a more profound reduction in serum TC compared with patients who were not treated. A similar decreasing trend was observed for serum LDL-C and non-HDL-C, whereas serum TG and HDL-C were not significantly affected by LT_4_ (Table 3). Glucose metabolism was not altered by LT_4_ supplementation given that FPG was the same at baseline and end-of-study within both groups.

## 4. Discussion

To our knowledge, this study is the first to evaluate the efficacy of LT_4_ replacement on NAFLD in SCH patients. We reported here that LT_4_ replacement treatment resulted in a reduction in the prevalence of NAFLD in significant SCH patients. Although we were not able to prove remarkable benefits of LT_4_ treatment on the prevalence of NAFLD and serum liver enzymes in mild SCH patients, subgroup analysis revealed that mild SCH patients with dyslipidemia could benefit from LT_4_ supplementation with profound reductions in the prevalence of NAFLD and serum liver enzymes. We also reported in the subgroup analysis that patients who received LT_4_ treatment exhibited trends of higher NAFLD remission rates and lower NAFLD incidence rates compared with patients who were not treated, albeit not statistically significant.

A number of studies have provided evidence supporting the role of SCH as an independent risk factor of NAFLD [13–16]. The mechanism underlying the association of SCH and NAFLD may be mediated by the direct regulation of TSH on liver lipid metabolism. Our group first proved that hepatocytes had functional TSH receptor (TSHR) expression, and the expression was not a case of illegitimate transcription [17]. Subsequently, we further demonstrated that TSH, by binding to TSHR on hepatocytes, upregulated the expression of hepatic 3-hydroxy-3-methyl-glutaryl coenzyme A reductase (HMGCR), which is the rate-limiting enzyme in cholesterol synthesis, and subsequently increased the cholesterol content in the liver [19]. In addition to the regulation of TSH on liver cholesterol metabolism, we also discovered a role of TSH in regulating triglyceride metabolism. We observed that TSH could aggravate the triglyceride accumulation in hepatocytes induced by a high-fat diet in mice through the activation of sterol regulatory element binding protein 1c (SREBP-1c), and TSHR knockout mice (*Tshr−/−*) exhibited a relatively lower degree of liver steatosis [18]. The present study was based on SCH patients and demonstrated that normalizing the elevated level of TSH in significant SCH patients or mild SCH patients with dyslipidemia via LT_4_ replacement therapy could reduce the prevalence of NAFLD. The results of this study were consistent with previous studies that revealed the independent role of elevated TSH in the course of NAFLD. This finding suggests that, when engaged with NAFLD patients, clinicians should be alert to the possibility of combination of SCH. For NAFLD patients suspected of secondary to SCH, normalizing thyroid function by appropriate supplementation with LT_4_ may be an effective means for controlling NAFLD.

Several lines of evidence have indicated that SCH is associated with dyslipidemia. The prevalence of dyslipidemia is higher in SCH patients compared with euthyroid subjects [35, 36]. Moreover, serum TSH levels are positively associated with serum lipid profiles independent of thyroid hormones [22]. In this study, the prevalence of dyslipidemia was as high as 62.7% in mild SCH patients, which is considerably increased compared with that reported in general Chinese population (estimated as 30% to 50%) [37, 38]. On the other hand, dyslipidemia is a risk factor associated with NAFLD [24–26]. A cross-sectional, community-based study conducted in Taiwan reported that elevated serum TG levels greater than 150 mg/dL were associated with an increased risk for NAFLD (odds ratio, 2.48; 95% confidence interval, 1.42–4.32; *p* = 0.001) among nonobese subjects [25]. In this study, we performed subgroup analysis in mild SCH patients with dyslipidemia and reported a significant decline in prevalence of NAFLD in subjects who received LT_4_ treatment compared with the control group. The benefits of LT_4_ supplementation on NAFLD found in the subgroup analysis suggests that LT_4_ replacement therapy should be suggested for mild SCH patients with dyslipidemia.

Previously, abnormalities in serum TG were more closely associated with NAFLD than serum cholesterol [39]. In recent years, evidence has suggested that atherogenic cholesterols were related to NAFLD either. Chan et al. reported that a diet enriched with cholesterol was involved in and essential for the development of liver steatosis in animal models [40]. Additionally, disturbances in liver cholesterol metabolism were common among NAFLD patients, suggesting a crucial role of cholesterol metabolism in the progression of NAFLD [41]. Furthermore, a prospective cohort study has indicated that non-HDL-C independently predicted new onset of NAFLD after adjusting for other confounders and was a stronger predictor for NAFLD than TG and LDL-C [42]. In this study, the subgroup analysis revealed that patients treated with LT_4_ experienced a significantly more profound reduction in serum TC compared with patients who were not treated, whereas serum TG levels were not significantly affected by LT_4_. Overall, we extrapolated that the decreased atherogenic cholesterol levels might contribute to the improvement of NAFLD.

Another interesting result of the subgroup analysis was that subjects who received LT_4_ exhibited a significant decrease in weight and BMI. Furthermore, patients who experienced remission of NAFLD exhibited greater decreases in body weight and BMI compared to those without remission of NAFLD. It has been well established that obesity has an independent effect on liver fat [43], and trial data have demonstrated that reduction in weight was associated with a significant improvement in the histological severity of NAFLD [8, 44]. It is plausible that LT_4_ supplementation could facilitate weight control by increasing the basal metabolic rate and subsequently ameliorating NAFLD. Further studies are needed to illuminate the precise mechanism under the benefits of LT_4_ supplementation on NAFLD in SCH patients.

There are some limitations of this study. First, this study was a retrospective post hoc analysis of the original randomized controlled trial; thus, the LT_4_ replacement therapy was not randomized in analyzed subjects. However, the subjects involved in this analysis were well balanced for baseline characteristics among treatment arms. Second, the diagnosis of NAFLD in this study was based on liver ultrasonographic examination. The optimal threshold for detecting steatosis on ultrasonographic imaging is about 30% in fat content [45, 46], which means that hepatic ultrasonography is not sufficiently sensitive to detect mild change in fatty infiltration. Also, ultrasonography has intrapractitioner variability in making a diagnosis. Since the trial was conducted in the rural area, we chose ultrasonography as the preferred method for diagnosis of the fatty liver given its advantage of being widely available and cost effective. Another limitation was that most of the patients in this study exhibited normal serum liver enzymes at enrollment. As a consequence, although we observed statistically significant reductions in serum liver enzymes in LT_4_-treated SCH patients, the reductions were relatively minor. Well-conducted trials with histological endpoints can provide more profound evidence of the benefits of LT_4_ on NAFLD.

## 5. Conclusions

In conclusion, this study demonstrated a beneficial effect of LT_4_ replacement therapy on NAFLD in patients with SCH, especially in significant SCH patients or mild SCH patients with dyslipidemia, with a decrease in the prevalence of NAFLD and serum liver enzymes. Our findings support the independent role of SCH for NAFLD and suggest the importance of early detection of thyroid function in patients with NAFLD. Moreover, based on our results, treatment of SCH with appropriate LT_4_ supplementation may be an effective means for controlling NAFLD. Double-blind, placebo-controlled trials with histological endpoints are needed to further validate the clinical benefits of LT_4_ supplementation on NAFLD.

## Supplementary Material

The information of supplementary materials are as follows: S1 file. Sup. Table 1 Efficacy of LT4 on Thyroid Function; S2 file. Changes in Metabolic Characteristics in the Cohort; S3 file. Sup. Table 4 Baseline characteristics for subgroup analysis; S4 file. Sup. Table 5 Number of NAFLD in mild SCH patients with dyslipidemia.

## Figures and Tables

**Table 1:** Baseline characteristics of participants.

Variables	Significant SCH-LT_4_ (*n* = 33)	Mild SCH patients		
		LT_4_ group (*n* = 181)	Control group (*n* = 149)	*p* value^∗^
Age (year)	57.79 ± 8.51	55.04 ± 7.85	56.59 ± 8.31	0.074
Female, *n* (%)	27 (81.8)	135 (74.6)	115 (77.2)	0.584
BMI (kg/m^2)^	25.88 ± 3.15	26.12 ± 3.11	25.72 ± 3.58	0.372
Weight (kg)	66.13 ± 10.26	66.46 ± 10.40	65.17 ± 11.24	0.308
WC (cm)	91.53 ± 9.17	90.81 ± 9.44	91.19 ± 10.52	0.675
ALT (IU/L)	18.61 ± 7.67	19.09 ± 9.00	17.20 ± 8.35	0.050
AST (IU/L)	26.12 ± 5.83	26.08 ± 6.80	24.65 ± 5.77	0.057
TC (mmol/L)	5.68 ± 0.93	5.91 ± 1.13	5.70 ± 1.05	0.086
HDL-C (mmol/L)	1.35 ± 0.27	1.41 ± 0.33	1.41 ± 0.32	0.923
LDL-C (mmol/L)	3.37 ± 0.91	3.40 ± 0.82	3.29 ± 0.80	0.222
Non-HDL-C (mmol/L)	4.33 ± 0.92	4.50 ± 1.09	4.29 ± 1.02	0.071
TG (mmol/L)	1.35 (0.78)	1.37 (1.00)	1.38 (1.02)	0.712
SBP (mmHg)	149.13 ± 19.61	149.92 ± 19.81	151.75 ± 23.80	0.435
DBP (mmHg)	82.16 ± 10.34	85.21 ± 11.66	86.01 ± 11.77	0.519
FPG (mmol/L)	6.98 ± 2.93	6.28 ± 1.68	6.34 ± 1.88	0.799
Obesity, *n* (%)				0.440
Normal (BMI< 24)	10 (30.3)	46 (25.4)	46 (30.9)	
Overweight (24 ≤ BMI< 28)	14 (42.4)	92 (50.8)	66 (44.3)	
Obese (BMI ≥ 28)	9 (27.3)	43 (23.8)	37 (24.8)	
Dyslipidemia, *n* (%)	22 (66.7)	116 (64.1)	91 (61.1)	0.573
NAFLD, *n* (%)	16 (48.5)	80 (44.2)	59 (39.6)	0.400
Diabetes, *n* (%)	9 (27.3)	25 (13.8)	25 (16.8)	0.455

Values for quantitative data are expressed as mean ± standard deviation or median (interquartile range); values for categorical variables are expressed as number (percentage).

^∗^ *p* value for comparing variables between mild SCH-LT_4_ group and mild SCH-control group.

SCH: subclinical hypothyroidism; LT_4_: levothyroxine; BMI: body mass index; WC: waist circumference; ALT: alanine aminotransferase; AST: aspartate aminotransferase; TC: total cholesterol; HDL-C: high-density lipoprotein cholesterol; LDL-C: low-density lipoprotein cholesterol; non-HDL-C: nonhigh-density lipoprotein cholesterol; TG: triglyceride; SBP: systolic blood pressure; DBP: diastolic blood pressure; FPG: fasting plasma glucose; NAFLD: nonalcoholic fatty liver disease.

**Table 2:** Prevalence of NAFLD and serum liver enzymes throughout the study in SCH patients.

Variables	Significant SCH-LT_4_ (*n* = 33)	Mild SCH patients		
		LT_4_ group (*n* = 181)	Control group (*n* = 149)	*p* value^∗^
Prevalence of NAFLD, *n* (%)				
Baseline	16 (48.5)	80 (44.2)	59 (39.6)	0.400
End-of-study	8 (24.2)	65 (35.9)	52 (34.9)	0.848
*p* value^#^	0.041	0.108	0.402	—
ALT (IU/L)				
Baseline	18.61 ± 7.67	19.09 ± 9.00	17.20 ± 8.35	0.050
End-of-study	17.15 ± 7.34	17.95 ± 7.71	17.29 ± 7.68	0.410
*p* value^#^	0.383	0.087	0.906	—
Change in ALT	−1.46 ± 9.44	−1.14 ± 8.77	0.09 ± 9.01	0.217
AST (IU/L)				
Baseline	26.12 ± 5.83	26.08 ± 6.80	24.65 ± 5.77	0.057
End-of-study	20.52 ± 6.69	21.22 ± 6.88	21.36 ± 6.48	0.854
*p* value^#^	<0.001	<0.001	<0.001	—
Change in AST	−5.61 ± 1.27	−4.86 ± 7.34	−3.29 ± 6.65	0.046

Values for quantitative data are expressed as mean ± standard deviation; values for categorical variables are expressed as number (percentage).

^∗^ *p* value for comparing variables between mild SCH-LT_4_ group and mild SCH-control group.

^#^ *p* value for comparing variables between baseline and end-of-study within each group.

NAFLD: nonalcoholic fatty liver disease; SCH: subclinical hypothyroidism; LT_4_: levothyroxine; ALT: alanine aminotransferase; AST: aspartate aminotransferase.

**Table 3:** Change from baseline in metabolic variables in mild SCH patients combined with dyslipidemia.

Variables	Sub-LT_4_ group (*n* = 116)			Subcontrol group (*n* = 91)			*p* value^∗^
	Baseline	End-of-study	*p* value^#^	Baseline	End-of-study	*p* value^#^	
BMI (kg/m^2)^	26.48 ± 3.13	26.00 ± 3.27	0.001	26.17 ± 3.68	26.20 ± 3.88	0.865	—
Weight (kg)	66.92 ± 10.27	65.44 ± 9.97	<0.001	66.12 ± 11.57	66.12 ± 12.03	0.993	—
TC (mmol/L)	6.27 ± 1.21	5.69 ± 1.15	<0.001	5.99 ± 1.16	5.74 ± 1.14	0.026	—
HDL-C (mmol/L)	1.33 ± 0.36	1.31 ± 0.31	0.301	1.31 ± 0.34	1.42 ± 0.37	<0.001	—
LDL-C (mmol/L)	3.67 ± 0.83	3.43 ± 0.91	<0.001	3.55 ± 0.85	3.35 ± 0.94	0.015	—
Non-HDL-C (mmol/L)	4.93 ± 1.08	4.37 ± 1.03	<0.001	4.68 ± 1.04	4.32 ± 1.02	0.001	—
TG (mmol/L)	1.82 (1.27)	1.49 (1.04)	<0.001	1.84 (1.29)	1.46 (0.90)	<0.001	—
FPG (mmol/L)	6.43 ± 1.54	6.35 ± 1.83	0.329	6.65 ± 2.04	6.50 ± 2.07	0.229	—
Change in TC		−0.58 ± 0.92			−0.25 ± 1.06		0.018
Change in LDL		−0.25 ± 0.63			−0.20 ± 0.77		0.595
Change in Non-HDL-C		−0.56 ± 0.84			−0.36 ± 0.95		0.102
Change in TG		−0.32 (0.88)			−0.34 (0.85)		0.392

Values are expressed as mean ± standard deviation or median (interquartile range).

^∗^ *p* value for comparing variables between sub-LT_4_ group and subcontrol group.

^#^ *p* value for comparing variables between baseline and end-of-study within each group.

SCH: subclinical hypothyroidism; LT_4_: levothyroxine; BMI: body mass index; FPG: fasting plasma glucose; TC: total cholesterol; HDL-C: high-density lipoprotein cholesterol; LDL-C: low-density lipoprotein cholesterol; non-HDL-C: nonhigh-density lipoprotein cholesterol; TG: triglyceride.
