# Supplementary material for: Benefits of Levothyroxine Replacement Therapy on Nonalcoholic Fatty Liver Disease in Subclinical Hypothyroidism Patients
Source: Int J Endocrinol. 2017 Apr 4;2017:5753039. doi: 10.1155/2017/5753039 (PMC5394912; doi:10.1155/2017/5753039)
Supplement: Supplementary file 1 — The information of supplementary materials are as follows: S1 file. Sup. Table 1 Efficacy of LT4 on Thyroid Function; S2 file. Changes in Metabolic Characteristics in the Cohort; S3 file. Sup. Table 4 Baseline characteristics for subgroup analysis; S4 file. Sup. Table 5 Number of NAFLD in mild SCH patients with dyslipidemia. [file 5753039.f1.doc]

Supplementary Table 1: Thyroid function throughout the study in SCH patients.

| Variables | Significant SCH- LT_4_  (*n =* 33) | Mild SCH patients | | |
| --- | --- | --- | --- | --- |
|  |  | LT_4_ group  (*n =* 181) | Control group  (*n =* 149) | *p* value* |
| TSH (mIU/L) | | | | |
| Baseline | 11.58 (1.96) | 5.98 (2.30) | 5.50 (1.85) | 0.138 |
| End-of-study | 1.86 (2.62) | 2.81 (1.44) | 4.90 (2.30) | < 0.001 |
| *P*-value**^#^** | < 0.001 | < 0.001 | < 0.001 | — |
| FT_4_ (pmol/L) | | | | |
| Baseline | 14.15 ± 1.37 | 14.87 ± 1.69 | 14.78 ± 1.71 | 0.680 |
| End-of-study | 19.01 ± 2.63 | 16.80 ± 2.35 | 14.89 ± 2.32 | < 0.001 |
| *P*-value**^#^** | < 0.001 | < 0.001 | 0.490 | — |
| FT_3_ (pmol/L) | | | | |
| Baseline | 4.88 ± 0.54 | 5.04 ± 0.56 | 4.97 ± 0.55 | 0.306 |
| End-of-study | 4.75 ± 0.74 | 4.98 ± 0.58 | 4.92 ± 0.67 | 0.103 |
| *P*-value**^#^** | 0.364 | 0.162 | 0.121 | — |

Values for are expressed as mean ± standard deviation, or median (inter-quartile range).

******p* value for comparing variables between mild SCH-LT_4_ group and mild SCH-Control group.

**^#^** *p* value for comparing variables between baseline and end-of-study within each group.

SCH, subclinical hypothyroidism; LT_4_, levothyroxine; FT_3_, free triiodothyronine; FT_4_, free thyroxine; TSH, thyroid-stimulating hormone.
