# Supplementary material for: Benefits of Levothyroxine Replacement Therapy on Nonalcoholic Fatty Liver Disease in Subclinical Hypothyroidism Patients
Source: Int J Endocrinol. 2017 Apr 4;2017:5753039. doi: 10.1155/2017/5753039 (PMC5394912; doi:10.1155/2017/5753039)
Supplement: Supplementary file 2 [file 5753039.f2.doc]

**Supplementary Results**

*1. Changes in metabolic characteristics in significant SCH patients*

After treated with LT_4_, the significant SCH patients experienced significant decrease in serum atherogenic cholesterols, including TC, LDL-C, and non HDL-C (*p* < 0.05 for all), whereas the serum HDL-C level and serum TG level were not significantly changed. The body weight of the significant SCH patients showed a trend of decrease, but did not reach the significant level (66.13 vs*.* 64.98 kg, *p* = 0.093) (Supplementary Table 2).

*2. Changes in metabolic characteristics in mild SCH patients combined with NAFLD who were treated with LT_4_*

As shown in the Supplementary Table 3, after treated with LT_4_, mild SCH patients that combined with NAFLD experienced profound reduction in serum AST level. Although there was a trend of decrease in serum ALT level, the decrease did not reach the significance level. Regarding serum lipids, serum TC, LDL-C, non HDL-C, and TG showed significant decrease from baseline to the end-of-study (*p* < 0.05 for all), but the serum HDL-C level was not significantly changed. Statistically significant reductions (*p* < 0.05) in mean body weight and BMI were observed in these patients.

Supplementary Table 2: Change from baseline in body weight and serum lipids in significant SCH patients (*n =* 33).

| Variables | Baseline | End-of-study | *p* value |
| --- | --- | --- | --- |
| BMI (kg/m^2^) | 25.87 ± 3.15 | 25.43 ± 3.15 | 0.144 |
| Weight (kg) | 66.13 ± 10.26 | 64.98 ± 9.55 | 0.093 |
| TC (mmol/L) | 5.68 ± 0.93 | 5.21 ± 0.89 | 0.008 |
| HDL-C (mmol/L) | 1.35 ± 0.27 | 1.31 ± 0.28 | 0.213 |
| LDL-C (mmol/L) | 3.37 ± 0.91 | 3.06 ± 0.66 | 0.028 |
| Non HDL-C (mmol/L) | 4.33 ± 0.92 | 3.90 ± 0.88 | 0.009 |
| TG (mmol/L) | 1.35 (0.68) | 1.32 (1.00) | 0.396 |

Values are expressed as mean ± standard deviation, or median (inter-quartile range).

BMI, body mass index; TC, total cholesterol; HDL-C, high-density lipoprotein cholesterol; LDL-C, low-density lipoprotein cholesterol; Non HDL-C, non high-density lipoprotein cholesterol; TG, triglyceride

Supplementary Table 3: Change from baseline in metabolic variables in mild SCH patients combined with NAFLD who were treated with LT_4_ (*n =* 80).

| Variables | Baseline | End-of-study | *p* value |
| --- | --- | --- | --- |
| ALT (IU/L) | 21.43 ± 10.32 | 20.13 ± 9.18 | 0.271 |
| AST (IU/L) | 26.28 ± 7.03 | 21.63 ± 8.52 | < 0.001 |
| TC (mmol/L) | 5.90 ± 1.15 | 5.46 ± 1.17 | < 0.001 |
| HDL-C (mmol/L) | 1.27 ± 0.29 | 1.26 ± 0.27 | 0.466 |
| LDL-C (mmol/L) | 3.46 ± 0.84 | 3.26 ± 0.93 | 0.007 |
| Non HDL-C (mmol/L) | 4.63 ± 1.06 | 4.20 ± 1.06 | < 0.001 |
| TG (mmol/L) | 1.82 (1.32) | 1.55 (1.16) | 0.004 |
| BMI (kg/m^2^) | 28.00 ± 2.77 | 27.61 ± 2.68 | 0.023 |
| Weight (kg) | 71.49 ± 9.51 | 70.24 ± 9.56 | 0.007 |

Values are expressed as mean ± standard deviation, or median (inter-quartile range).

ALT, alanine aminotransferase; AST, aspartate aminotransferase; BMI, body mass index; TC, total cholesterol; HDL-C, high-density lipoprotein cholesterol; LDL-C, low-density lipoprotein cholesterol; Non HDL-C, non high-density lipoprotein cholesterol; TG, triglyceride.
