# Supplementary material for: Benefits of Levothyroxine Replacement Therapy on Nonalcoholic Fatty Liver Disease in Subclinical Hypothyroidism Patients
Source: Int J Endocrinol. 2017 Apr 4;2017:5753039. doi: 10.1155/2017/5753039 (PMC5394912; doi:10.1155/2017/5753039)
Supplement: Supplementary file 3 [file 5753039.f3.doc]

Supplementary Table 4: Baseline characteristics for mild SCH patients in combination with dyslipidemia.

| Variables | Sub-[LT_4_](app:ds:Euthyrox) group  (*n* = 116) | Sub-Control group  (*n* = 91) | *p* value* |
| --- | --- | --- | --- |
| Age (year) | 56.25 ± 7.28 | 56.92 ± 8.20 | 0.533 |
| Female, n (%) | 89 (76.7) | 72 (79.1) | 0.681 |
| BMI (kg/m^2)^ | 26.48 ± 3.13 | 26.17 ± 3.68 | 0.616 |
| Weight (kg) | 66.92 ± 10.27 | 66.12 ± 11.57 | 0.620 |
| WC (cm) | 91.92 ± 9.44 | 93.14 ± 10.38 | 0.368 |
| ALT (IU/L) | 19.93 ± 10.60 | 17.59 ± 7.67 | 0.099 |
| AST (IU/L) | 26.70 ± 7.49 | 24.68 ± 5.91 | 0.052 |
| TC (mmol/L) | 6.27 ± 1.21 | 5.99 ± 1.16 | 0.099 |
| HDL-C (mmol/L) | 1.33 ± 0.36 | 1.31 ± 0.34 | 0.684 |
| LDL-C (mmol/L) | 3.67 ± 0.83 | 3.55 ± 0.85 | 0.257 |
| Non HDL-C (mmol/L) | 4.93 ± 1.08 | 4.68 ± 1.04 | 0.087 |
| TG (mmol/L) | 1.50 (1.04) | 1.46 (0.90) | 0.821 |
| SBP (mmHg) | 152.44 ± 18.56 | 154.11 ± 24.21 | 0.604 |
| DBP (mmHg) | 86.08 ± 11.50 | 87.48 ± 12.04 | 0.415 |
| FPG (mmol/ L) | 6.43 ± 1.54 | 6.65 ± 2.04 | 0.390 |
| Obesity, n (%) |  |  | 0.722 |
| Normal (BMI < 24) | 25 (21.6) | 24 (26.0) |  |
| Overweight (24 ≤ BMI < 28) | 58 (50.0) | 42 (45.7) |  |
| Obese (BMI ≥ 28) | 33 (28.4) | 26 (28.3) |  |

Values for quantitative data are expressed as mean ± standard deviation, or median (inter-quartile range); values for categorical variables are expressed as number (percentage).

******p* value for comparing variables between sub-LT_4_ group and sub-Control group.

SCH, subclinical hypothyroidism; LT_4_, levothyroxine; BMI, body mass index; WC, waist circumference; ALT, alanine aminotransferase; AST, aspartate aminotransferase; TC, total cholesterol; HDL-C, high-density lipoprotein cholesterol; LDL-C, low-density lipoprotein cholesterol; Non HDL-C, non high-density lipoprotein cholesterol; TG, triglyceride; SBP, systolic blood pressure; DBP, diastolic blood pressure; FPG, fasting plasma glucose.
