# Supplementary material for: Benefits of Levothyroxine Replacement Therapy on Nonalcoholic Fatty Liver Disease in Subclinical Hypothyroidism Patients
Source: Int J Endocrinol. 2017 Apr 4;2017:5753039. doi: 10.1155/2017/5753039 (PMC5394912; doi:10.1155/2017/5753039)
Supplement: Supplementary file 4 [file 5753039.f4.doc]

Supplementary Table 5: Number of subjects that diagnosed with NAFLD in mild SCH patients with dyslipidemia.

| Sub-[LT_4_](app:ds:Euthyrox) group (*n* = 116) | | | | |  | Sub-Control group (*n* = 91) | | | | |
| --- | --- | --- | --- | --- | --- | --- | --- | --- | --- | --- |
| Baseline | |  | End-of-study | |  | Baseline | |  | End-of-study | |
| NAFLD | n |  | NAFLD | n (%) |  | NAFLD | n |  | NAFLD | n (%) |
| No | 53 |  | No | 43 (81.1) |  | No | 51 |  | No | 41 (80.4) |
|  |  |  | Yes | 10 (18.9) |  |  |  |  | Yes | 10 (19.6) |
| Yes | 63 |  | No | 26 (41.3) |  | Yes | 40 |  | No | 14 (35.0) |
|  |  |  | Yes | 37 (58.7) |  |  |  |  | Yes | 26 (65.0) |

NAFLD, non-alcoholic fatty liver disease; LT_4_, levothyroxine; SCH, subclinical hypothyroidism.
